# Supplementary material for: Chromothripsis during telomere crisis is independent of NHEJ, and consistent with a replicative origin
Source: Genome Res. 2019 May;29(5):737–49. doi: 10.1101/gr.240705.118 (PMC6499312; doi:10.1101/gr.240705.118)
Supplement: Supplemental Material [file supp_gr.240705.118_Supplemental_file_1.zip › contigs/annotated_contigs/DB77/contig.2.DB77_length_680_mean_cov_8.75294117647.docx]

**DB77_length_680_mean_cov_8.75294117647**

```
TCGGAGCACATCTGGTGCGAGGACTTTCTGGTGCGGAGCTTCTACCTGAAGAACGTGCAGACCCAGGAGACGCGCACGCTCACGCAGTT
 >chr2:220159531-220159851 - E=9e-181
CCACTTCCTCAGCTGGCCGGCAGAGGGCACACCGGCCTCCACGCGGCCCCTGCTGGACTTCCGCAGGTGAGCGCGGGCGCCCCCGCCTG

GAGGGACCGCATGGGCGGGGGCGGAGCCGGGGTCCGCCCGCCGCTCGGCCTGGAGCCCCGGGTCGGGCCTGGAGCCTCGGCCTCTGACT

TCTGGCCGCCCGCTCCAGACCCCACGCTCCCACCCCTAAAAGCAGGCCTG|AGG|GCTCTTTCTTGCTTTCTCCCCATTCCATATATTG
                                                   >chr2:220583046-220583409 - E=1e-203
TCACTTTTTCCTTAATGAGAACCCTGCTTCCCAATAGTAACACATTTACTTATTTGCTCACTCCTATAATACATATGTCAAACCTAAAT

AACAGACAGAGTGGGAGACTCTCTAAAAGAAAATGATATTTATTAAGGAGTAGCATTGCAATGGGAATGCACATGCCATAGTAAACTAT

GTGCCTATTCAGGGAGGTTGAGACAAATGGAAGTTTTTAAAGGAAAAATGAGGGGGATTACATAATTTGCTTTGAGTCAATTATCCTTG

GCTACAAGGTTCAGTAACAAGGGTGGTGTCAGTCTGAGGTTGGATGGGCAGTTGCTGAA
```
